# Inducible Treg cell populations as cell based-therapy for rheumatoid arthritis

**DOI:** 10.1186/1479-5876-10-S3-P55

**Published:** 2012-11-28

**Authors:** Delphine Martire, Julie Quentin, Anne-Laure Mausset-Bonnefont, Hélène Asnagli, Nathalie Belmonte, Arnaud Foussat, Christian Jorgensen, Pascale Louis-Plence

**Affiliations:** 1 Inserm U844, Université Montpellier 1, CHU Lapeyronnie, Montpellier, France; 2 TxCell, Valbonne-Sophia Antipolis, France

## Background

Adoptive cell transfer of Treg cells is a promising approach to restore tolerance in autoimmune disease.

However the various type of Tregs, their doses of injection and their *in vivo*-suppressive mechanism need to be precisely define to clearly establish which Tregs will be able to dampen efficiently the immune response in the various settings.

In our study, we compared the therapeutic potential of induced CD25^+^FoxP3^+^ and two IL10-secreting Tregs: Tr1 and CD49b-induced Tregs.

## Materials and methods

CD49b Treg cells were generated in naive mice following repetitive injections of iDC. The purification was based on the negative selection of CD4 T cells isolated from the spleen and liver of the iDC-vaccinated mice.

Cell sorting experiments were realized to obtain 98% pure CD49b^+^ T or CD25^+^ cells. Collagen type II (bCII) specific Tr1 clones were obtained from TCR transgenic mice and expanded *in vitro*. Selected clones showed *in vitro* antigen specificity, Tr1 cytokine profile and IL10- and TGFβ-dependent suppressive activity.

## Results

Several doses of CD49b or Tr1 cells were injected i.v. at day 28 in established collagen-induced arthritis. Clinical signs of arthritis were scored, as well as biological parameters such as the level of anti-bCII antibodies in sera and the cytokine profile of bCII specific T cells.

We defined for both Treg cell populations the dose effect in curative settings experiments. One single dose of 3x10^6^ or 1x10^6^ of Tr1 cell administration could reduce the incidence and severity of CIA. Interestingly, higher dose of 10M of Tr1 cells did not improve the disease. In the same manner, the dose of 10^5^ CD4CD49b^+^ or CD25^+^ cells reverse clinical symptom with a lack of efficacy of higher doses. The cytokinic profile of the Tr1 cells was also investigated in inflammatory settings as well as the impact of the various Treg cells on the proliferation of effector cells *in vivo*.

## Conclusions

Our results suggest that even if the Treg cells present some similarities, we need to precisely define the dose and type of Treg that will be efficient in each experimental setting.

